# Colony-Level Effects of Amygdalin on Honeybees and Their Microbes

**DOI:** 10.3390/insects11110783

**Published:** 2020-11-11

**Authors:** James P. Tauber, Cansu Ö. Tozkar, Ryan S. Schwarz, Dawn Lopez, Rebecca E. Irwin, Lynn S. Adler, Jay D. Evans

**Affiliations:** 1 Bee Research Laboratory, Beltsville Agricultural Research Center, US Department of Agriculture, Beltsville, MD 20705, USA; tozkar@gmail.com (C.Ö.T.); rsschwarz@fortlewis.edu (R.S.S.); dawn.lopez@usda.gov (D.L.); 2 Department of Agricultural Biotechnology, Faculty of Agriculture, Yüzüncü Yıl University, Van 65000, Turkey; 3 Department of Biology, Fort Lewis College, 1000 Rim Drive, Durango, CO 81301, USA; 4 Department of Applied Ecology, North Carolina State University, Raleigh, NC 27695, USA; reirwin@ncsu.edu; 5 Department of Biology, University of Massachusetts, Amherst, MA 01003, USA; lsadler@bio.umass.edu

**Keywords:** honeybees, natural products, amygdalin, microbes, viruses

## Abstract

**Simple Summary:**

Nectar compounds have the potential to affect microbial communities and pollinator immunity. Here, we investigated how the almond compound, amygdalin, influences the microbial community of the western honeybee. Using RNA sequencing technology to count microbial reads and bee gene transcripts, we show relatively no large change of bacterial counts, fungal counts or bee transcripts due to amygdalin treatment at the colony level. Larger fluctuations, perhaps due to amygdalin, were observed for pathogenic viruses and the pathogen *Lotmaria passim*; however, these changes could have been seasonal. Overall, amygdalin consumption at field-relevant, colony-levels may not have a large impact on bee symbionts or immune gene expression.

**Abstract:**

Amygdalin, a cyanogenic glycoside, is found in the nectar and pollen of almond trees, as well as in a variety of other crops, such as cherries, nectarines, apples and others. It is inevitable that western honeybees (*Apis mellifera*) consistently consume amygdalin during almond pollination season because almond crops are almost exclusively pollinated by honeybees. This study tests the effects of a field-relevant concentration of amygdalin on honeybee microbes and the activities of key honeybee genes. We executed a two-month field trial providing sucrose solutions with or without amygdalin *ad libitum* to free-flying honeybee colonies. We collected adult worker bees at four time points and used RNA sequencing technology and our HoloBee database to assess global changes in microbes and honeybee transcripts. Our hypothesis was that amygdalin will negatively affect bee microbes and possibly immune gene regulation. Using a log_2_ fold-change cutoff at two and intraday comparisons, we show no large change of bacterial counts, fungal counts or key bee immune gene transcripts, due to amygdalin treatment in relation to the control. However, relatively large titer decreases in the amygdalin treatment relative to the control were found for several viruses. Chronic bee paralysis virus levels had a sharp decrease (−14.4) with titers then remaining less than the control, Black queen cell virus titers were lower at three time points (<−2) and Deformed wing virus titers were lower at two time points (<−6) in amygdalin-fed compared to sucrose-fed colonies. Titers of *Lotmaria passim* were lower in the treatment group at three of the four dates (<−4). In contrast, Sacbrood virus had two dates with relative increases in its titers (>2). Overall, viral titers appeared to fluctuate more so than bacteria, as observed by highly inconstant patterns between treatment and control and throughout the season. Our results suggest that amygdalin consumption may reduce several honeybee viruses without affecting other microbes or colony-level expression of immune genes.

## 1. Introduction

Given the value of pollinators in agriculture coupled with declining populations, due to a variety of stressors, including pathogens, it is important to understand how natural plant products found in nectar impact pollinator health and immunity [1,2,3]. Model systems ideal for studying these relationships are the western honeybee (*Apis mellifera*) and bumblebee species (*Bombus terrestris* and *B. impatiens*). Pathogens that affect bees have been correlated with honeybee colony loss [4]. Key agents include Deformed wing virus (DWV; a single-stranded RNA virus), *Nosema ceranae* (an intracellularly reproducing fungal spore-producing parasite), and *Lotmaria passim* (eukaryotic trypanosomatid) [5,6]. Conversely, numerous microorganisms benefit honeybee health and immunity, such as bacterial mutualists found in the intestine, including *Snodgrassella alvi*, *Gilliamella apicola*, and *Lactobacillus* spp. [7]. The role of *Frischella perrara* is less clear [8].

Numerous studies have shown how natural plant products, specifically phytochemicals, can reduce pathogen loads in bees (reviewed [9]). For example, thymol (an essential oil from thyme plants, including in the nectar [10]) reduced *Nosema ceranae* spore loads and increased honeybee longevity relative to control worker bees [11]. Modeling data from feeding assays of phytochemicals during nosemosis showed that the interaction between concentration and compound was responsible for reduced spore counts at the end of the experiment [12]. In bumblebees, multiple nectar phytochemicals also reduced infection intensity by the gut pathogen *Crithidia bombi* [3,13]. Bees may forage on specific phytochemicals as a means of reducing colony pathogen loads. The notion of reducing pathogens by collecting plant resins or specific nectars has been considered a form of “social medication” [14]. While self-medication for bees is defined by the collection of remedies like phytochemicals to support individual health, social medication describes the collection of such remedies for the benefit of the colony [14]. As one example, honeybees will increase resin collection when the colony is infected with *A. apis* [15]. Additionally, at the colony level, collected plant resins reduce honeybee immune gene expression, which suggests that plant-derived compounds may reduce individual immunity costs [16,17]. Other potential benefits of phytochemicals may include improved memory and learning [18].

Despite these potential benefits, there may be costs and tradeoffs to collecting nectar with phytochemicals, especially when there are few nectar choices available, and those costs may be concentration- and species-dependent [19,20]. Costs may include both outright death [21], as well as sublethal ones. One example of a potential sublethal effect from a natural product is from thymol ingestion, where body mass was significantly reduced at non-lethal concentrations [22]. Despite an energetic cost, the collection of compounds may nonetheless be beneficial for the overall health of the colony [23]. In monoculture agricultural contexts, the consumption of specific nectar or pollen phytochemicals may be the only option for insects during specific times of the year, and it is important to know how this consumption impacts pollinator health and immunity.

The almond crop is worth billions of dollars per annum in the USA and Australia [24], and this commercial enterprise uses western honeybees as a major pollinator. Almond orchards (*Prunus dulcis*) are pollinated early in the year. Phytochemicals in almond nectar are collected by honeybees and consumed during the pollination season, potentially even if they are detrimental. Although it is possible that bees also forage on other plants during almond bloom, bees appear to have fidelity to forage on specific trees in orchards if the plant provides enough nutrition (reviewed in [25]). However, in another study, non-almond pollen was identified on bees foraging in almond orchards, presumably because bees were foraging on other plant species [25]. Thus, it is possible that bees have foraging options during almond bloom, but because almond is the major blooming floral resource and bees have fidelity towards crop trees, there is likely little potential for “social medication” or “social detoxification” (the latter by diluting toxic compounds through other foraging [26]).

Almond nectar and pollen contain amygdalin, a cyanogenic glycoside [27,28]. When amygdalin is broken down in animals, it forms cyanide, which is toxic to animals. However, despite its potential toxicity, honeybees can tolerate relatively high doses of amygdalin, as shown in survival assays where bees fed three log-scale concentrations of amygdalin, up to 100 ppm (*ad libitum*), had similar survival to the control for up to 22 days [29]. In another study using unnaturally high concentrations of amygdalin (up to 10 mM), the ingestion of amygdalin led to malaise signs, such as increased time spent upside down, as well as more abdomen dragging, but presumably, this was not lethal [30]. In a study using the bumblebee *B. impatiens* infected with the pathogen *Crithidia bombi,* amygdalin did not reduce pathogen titers nor increase mortality [3]. Natural concentrations of amygdalin average 4.9–6.7 ppm in *P. dulcis* nectar [27]. It has also been observed that bees do not avoid amygdalin at natural concentrations [31].

Since honeybees collect nectar and pollen containing amygdalin in almond orchards with likely only a few other major sources of nectar for dilution, we measured the effects of season-long amygdalin consumption on honeybee microbiota and immune gene expression. We hypothesized that season-long amygdalin ingestion would reduce honeybee microbial loads, which include parasites, viruses, mutualists and commensals. Although amygdalin’s derivative is considered toxic, animals can tolerate it, but we also hypothesized that microbes are less tolerant. Additionally, the toxin could harm host cells, which have intracellular pathogens. Other subtler host-pathogen-natural product interactions may also be at play. Understanding the effects of amygdalin on the bee microbiome and pathobiome will provide insight into other efforts for improving honeybee health using natural products [32], as well as our general understanding of mechanisms affecting pollinator health during commercial operations.

## 2. Materials and Methods

### 2.1. Experimental Setup and Compound Feeding

Twelve free-flying *A. mellifera* ligustica US domestic hybrid colonies located in a bee yard at the USDA in Beltsville, Maryland, were used. Colonies had been established from packages of honeybees delivered from a commercial breeder in Georgia, USA. We placed colonies on concrete blocks in a circular array with 2 m distance between colonies. In April 2013, we randomly assigned twelve healthy and similarly sized colonies into treatment and control groups, and then fed all colonies with sterile 50% sucrose-distilled water solutions placed in clean Mason jars with lids that had pin-sized punctured holes (Boardman hive-front feeders). The treatment group received this sucrose solution supplemented with 10 ppm (10 mg/L) dissolved amygdalin (Sigma, St. Louis, USA; CAS 29883-15-6). The control colonies received sucrose water. The amygdalin concentration was chosen to be slightly higher than in *P. dulcis* nectar (ca. 4.9–6.7 ppm [27]) under the assumption that these field colonies were also taking advantage of an active local nectar flow that might dilute amygdalin consumption. During this study, the mid-Atlantic region of the US is in full bloom [33]. Colonies were fed *ad libitum* throughout the experiment. The USDA-ARS apiary in Beltsville, Maryland, is not adjacent to any almond orchards. The experiment lasted approximately two months.

### 2.2. RNA Isolation and Sequencing

After two weeks of treatment and approximately every 14-18 days thereafter, approximately 80 adult bees were collected from each colony using a hand vacuum [32] to remove bees from the surface of populated brood frames. Four collection time points were made during the experiment. Total RNA was extracted from a pool of 50 bees per colony per time point using homemade RNA isolation buffer and phenol-chloroform, as described in the COLOSS BEEBOOK [34].

Total RNA was first quantified using a Nanodrop ND-8000 (ThermoFisher Scientific, Inc. Wilmington, DE, USA) with 2 μL of each sample (Supplementary Information). Additionally, RNA integrity was confirmed using a Bioanalyzer instrument. For each time point, we produced twelve total RNA extracts, one from each colony. Before RNA sequencing, we pooled the six total RNA samples from treated colonies at an equimolar concentration to produce one sample per time point per treatment for RNA sequencing. We did this separately for the six treatment colonies and the six control colonies. Library preparation and sequencing were done at the University of Maryland (UMD) Institute of Genome Sciences (IGS), Baltimore, Maryland. Library preparation was strand-unspecific and not a reduced representation. We produced 100 bp, paired-end data in an Illumina Hi-Seq 2000 machine [35]. The reads were received demultiplexed and trimmed of the adaptor.

### 2.3. Identification of Microbes

We ran the paired FASTQ files received from UMD in fastp for further quality control [36]. These FASTQ files were uploaded to the public NCBI SRA database (project PRJNA630027, containing files SRR11671127-34). Deposited samples were labeled consecutively as A1, A2, B1, B2, C1, C2, D1 and D2: for each time point (A (May 10, 2013); B (May 28, 2013); C (June 14, 2013); and D (July 1, 2013) with the amygdalin treatment (labeled with 1) and the control (labeled with 2). We aligned the paired-end, quality-controlled reads against an index of the *A. mellifera* genome (GCF_003254395.2_Amel_HAv3.1_genomic.fa [37]) using Hisat2 with default parameters (-1 -2 -S –dta) to remove honeybee reads (Supplementary Information). Reads that did not map to the bee genome were further processed for microbe titer analysis. We used samtools view (-S -f4 -h), flagstat, sort (-n), and fastq (-1 -2 -s -n) to extract the unmapped reads, check the file, order the reads and convert the file for Kraken2 input [38]. The unmapped, paired reads were then run in Kraken2 (2.0.8-beta) (--db –paired –report –report-zero-counts –use-names –confidence 0.04) to count microbes [39]. The Kraken2 database was built based on the organisms listed in our HoloBee dataset (https://data.nal.usda.gov/dataset/holobee-database-v20161) which included bacteria, fungi, metazoa, protozoa and viruses that are known associates of bees. To this end, we built the Kraken2 database by preferentially selecting one ‘representative genome’ for each species or genus. In some cases, we skipped a species or strain because no specific genome was available, or if the identity of the organism was ambiguous. Genomes are listed in Table S1. We added two yeasts that we have since isolated from honeybee intestines (*Metschnikowia reukaufii* (unpublished) and *Wickerhamomyces anomalus* [40]). Bracken [41] was then used on the Kraken2 output for count correction by read bp length.

Microbe and virus titers were examined in two ways. For the first method, which is only presented in Table 1 and used to evaluate how comprehensive our HoloBee-based search was, the Kraken-style Bracken output was modified to include the unclassified reads that were previously specified in the Kraken2 output. For each clade, these reads were divided by total unmapped bee reads that were available for microbe counting (i.e., microbe classified plus microbe unclassified reads) in each sample and multiplied by 100 to obtain a percentage of relative fragment coverage (Table 1; Table S2). Using this methodology, samples were essentially independent of one another with a normalization factor of 1. These results were compared to a run using a Kraken2 database built from NCBI’s RefSeq sequence data for bacteria, fungi and viruses. Comparison of the two runs showed similar classified percentages, except for fungi, for which the RefSeq database does not include *Nosema* (Table 1). This indicated that our HoloBee database captured most of the microbes and viruses in the samples because increasing the number of species from a reliable database did not clearly increase the number of hits.

**Figure 1:**
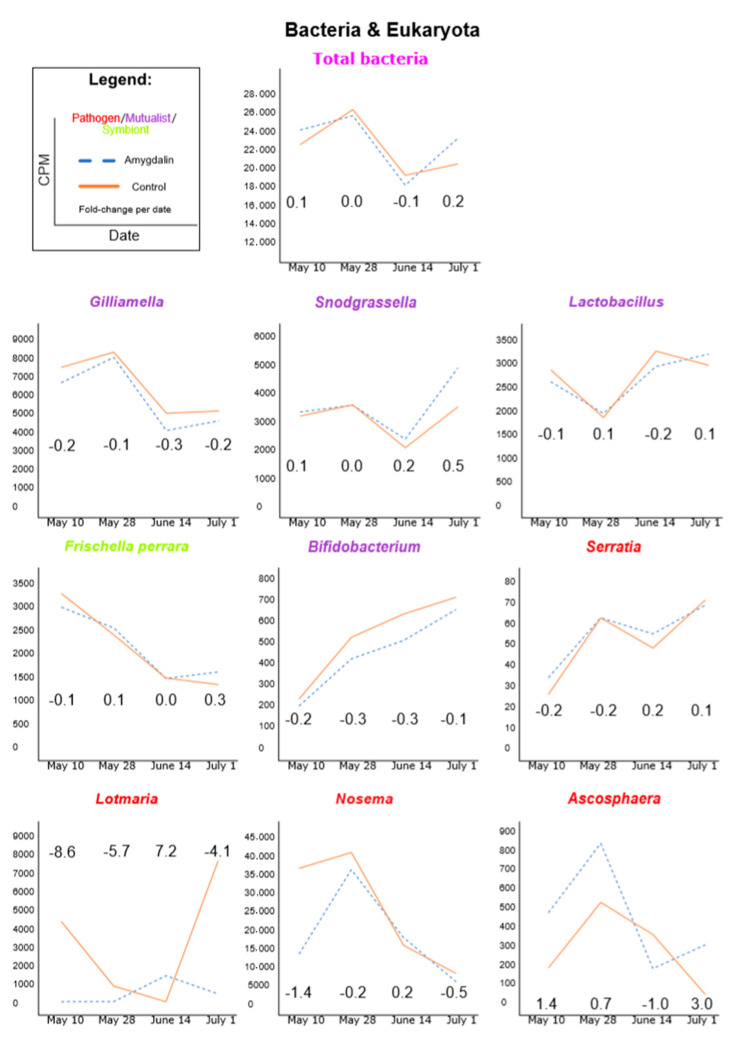
Bracken-corrected Kraken2 microbe counts (excluding viruses) using a Kraken2-HoloBee database. Counts were TMM normalized and presented as CPM (counts per million). Graphs were ordered from highest to lowest counts and by taxonomy. Numbers within the graph’s area indicate the log_2_ fold-change of amygdalin/control for that date. We considered a large increase in counts when the fold-change was greater than 2 and a large decrease when less than −2.

**Figure 2:**
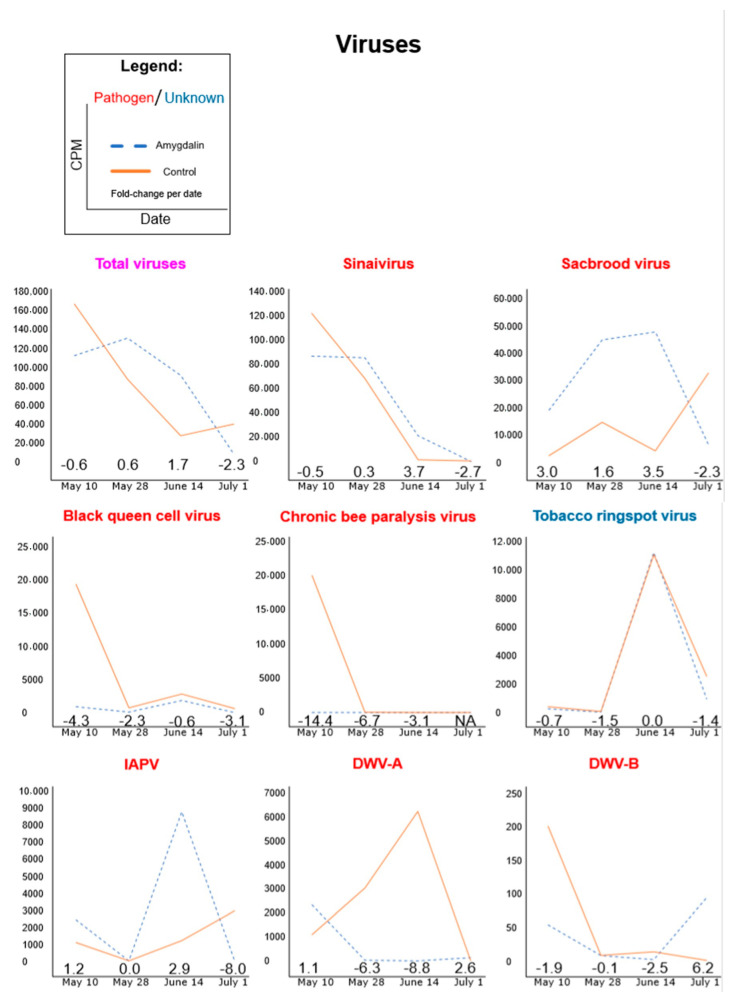
Bracken-corrected Kraken2 viral counts using a Kraken2-HoloBee database. Counts were TMM normalized and presented as CPM (counts per million). Graphs were ordered from highest to lowest counts. Numbers within the graph’s area indicate the log_2_ fold-change of amygdalin/control for that date. We considered a large increase in counts when the fold-change was greater than 2 and a large decrease when less than −2. Total viral titers appeared driven by Sinaivirus and Sacbrood virus.

In the second method, we imported the non-redundant clade counts (third column of the output) from the Kraken-style Bracken output into EdgeR within R Gui and normalized the samples together using TMM and CPM (Table S2, which contains the R script and library size adjustments) [42,43]. Estimating microbial abundances using the weighted trimmed mean of M-values and exporting them as count-per-million (CPM) was similar to previous honeybee microbe count work [44], except that we included unclassified microbe reads for the sample size adjustment. Although some assumptions of the TMM method are presumably not met, such that most microbes will have consistent abundance over time and microbes that are not consistent will have balanced count changes over time [45], we believe this method helps compensate for confounding factors, and thus, our interpretation of data was based on this second method (Figures 1 and 2).

Microsoft Excel, Inkscape (0.92.4; PC), and Pavian [46] (the latter using Rgui, 32-bit (https://cran.r-project.org/bin/windows/base/; PC; v3.6.3)) were used to view data and to produce graphics.

To supplement Kraken2, we prepared *de novo* assembled scaffolds. The .fastq paired (-1 and -2) and unpaired (-s) data from samtools, as described above, were *de novo* assembled using the script metaspades.py [47]. The scaffolds were run with BLASTN [48] (--remote) using an E-value threshold of 1e-100, megablast, the nt database and accepting/culling five hits (April 2020). We summarized microbe and virus hits after filtering out scaffolds less than 1 kb and looking for the highest percent identity (Table S3).

### 2.4. Gene Expression Analysis

**Figure 3:**
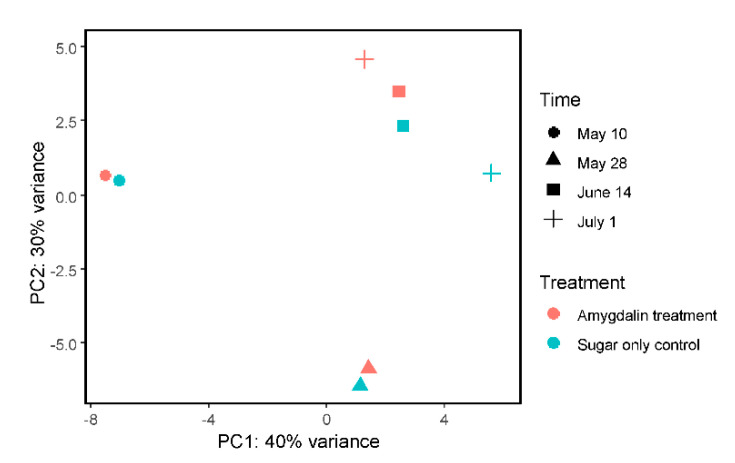
PCA plot using rlog-transformed data of known honeybee transcript counts from all RNAseq data for all four collection time points for both the amygdalin treatment and sugar-only control.

Using the reads that mapped to the bee genome from Hisat2, we used samtools to modify the .sam output using view (-S -b) and then sort. Stringtie [49] was then used (-e -o -G) with GCF_003254395.2_Amel_Hav3.1_genomic.gff to assemble transcripts and estimate coverage values. After each sample was independently run, we merged all transcripts using Stringtie merge. The files were then again run with Stringtie using the merged file as the reference (-G -b -o -A -e -C), limiting the search to only known transcripts. A gene count matrix was extracted from the Stringtie output using Stringtie’s script prepDE.py (-l 100 -i ./Stringtie.index). Counts were then imported into rGui for analyses using DESeq2 (as well as vsn, hexbin, pheatmap, rColorBrewer, and ggplot2) [50]. Phenotypic data were similarly imported and included treatment (amygdalin or control) and time point (A, B, C and D, as listed above). The design included treatment and time as variables. Because we only have one replicate per condition per time point, we did not consider doing any differential expression analysis (and estimation of dispersion would be misleading). To this end, we chose to look at FC (fold-change). Normalized log2 +1 was calculated within DESeq2 and exported to Excel (Table S4). This was merged with GCF_003254395.2_Amel_hAv3.1_feature_table.csv with readr in R. We sorted the table, and the fold-change was calculated as follows: log_2_FC = log_2_(amygdalin)– log_2_(control) and then FC = 2^log2FC^. We selected the top 20 to 40 up- and down-regulated genes from each time point. HymenopteraMine v1.4 [51] was used for GO term enrichment from these FC genes. For graphing Figure 3, we first transformed the data using rlog (blind=FALSE) to create a PCA plot (plotPCA) using these transformed data [52]. High performance computing servers were provided by the BAM (Berlin, Germany), and software was used in Gnome.

## 3. Results

### 3.1. Amygdalin Consumption May Change Certain Microbe and Viral Titers

In general, microbes and viruses fluctuated over time regardless of the treatment (Figure 1 and Figure 2). Our search used the Kraken2 algorithm and was limited to our curated HoloBee dataset, which includes microbes and viruses that have been isolated from *Apis* spp. Because we had only one pooled replicate per treatment group per time point, we did not conduct formal statistical analyses, but instead describe patterns below. We provide log_2_ fold-change (FC) as amygdalin/control with a cutoff of <−2.0 for relatively lower titers and >2 for relatively higher titers of amygdalin compared to the control within a specific date (as provided in parenthesis throughout the text). This corresponds to a fourfold increase or decrease, due to diet treatment.

For bacteria (Figure 1), when we apply our fold-change cutoff criteria, we observed no notable change in *Bifidobacterium asteroids,* Lactobacillales, *Gilliamella*, *Snodgrassella*, *Frischella perrara*, *Parasaccharibacter apium* nor *Serratia* *marcescens* loads in the amygdalin treatment relative to the control. However, we note certain trends. When we compare the overall seasonal patterns of *Gilliamella*, *Snodgrassella*, *Serratia* and total bacterial loads, they followed the same pattern, that is, each had similarly higher or lower titers on a specific date. Additionally, when looking at particular microbes, we saw that *Snodgrassella* had generally higher counts in the amygdalin treatment relative to the control throughout the season, which was the opposite for *Gilliamella* and *Bifidiobacterium*. Lastly, while *Snodgrassella* and *Gilliamella* titers fluctuated to higher and lower levels of overtime, *Bifidiobacterium* loads in both groups constantly increased over the summer, while *F. perrara* loads, for the most part, decreased over the summer.

For fungi and eukaryotes (Figure 1), when we apply our fold-change cutoff criteria, *L. passim* titers were lower in the amygdalin treatment relative to the control for three of the four dates (<−4), but one date had higher loads (7.2). We observed no notable changes in *Nosema* nor *Ascosphaera apis*, although at the last time point levels of *A. apis* increased in the amygdalin group (3.0). *Nosema* loads were relatively lower in three of the four time points in the amygdalin group, although not below the preset cutoff.

Several virus titers appeared to be reduced from amygdalin compared to control treatments, although this was not the case for every date (Figure 2). We observed lower DWV-A titers at two dates (<−6.3), DWV-B had one date with lower titers (−2.5), lower loads of Chronic bee paralysis virus at three dates (<−3.1), and lower Black queen cell virus loads at three dates (<−2.3). Conversely, higher titers in the amygdalin group relative to the control were found for Sacbrood virus titers at two dates (>3.0), Israeli acute paralysis virus (IAPV) at one date (2.9), and Sinaivirus at one date (3.7). Of all four dates, June 14 had the largest increase in total viral loads (1.7), with three of the seven key viruses having increased titers on this day. The last date (July 1) had a markedly lower total viral titers (−2.3), driven by Sacbrood virus (−2.3), Israeli acute paralysis virus (−8.0), Black queen cell virus (−3.1), and Sinaivirus (−2.7), except for DWV-A and DWV-B which had relatively higher titers (2.6 and 6.2, respectively). As a control, we also looked at the Tobacco ringspot virus, which is found in honeybees, but currently has no concrete evidence showing that it is adapted in the western honeybee [53,54]. There were no clear differences in the amygdalin and control treatment for the Tobacco ringspot virus count data, which was not the case for almost every other honeybee virus inspected.

*De novo* assembled scaffolds were used to support the presence of a microbe (Table S3). Although consistent taxonomic hits for scaffolds were not found across all eight samples, every HoloBee-oriented microbe of interest was identified in at least one sample through BLASTN, except for the following: bacteria (*P. apium* and *S. marcescens*); fungi (aspergilli and yeasts); other (*L. passim* and *T. mercedesae*). This could be due to our stringent megablast parameters, relatively lower abundance of these organisms or technical reasons, such as short 100 bp cDNA reads. Across the eight samples, the most consistently identified microbes were bacteria (*G. apicola* and *S. alvi*; found in all samples except one sample did not have a hit for *S. alvi*); fungi (*Nosema ceranae*, found in all but one sample); and viruses (Sacbrood virus, Black queen cell virus and Lake Sinai virus; found in all samples). Other viruses were identified, including *Apis rhabdovirus*, which coincides with a recent report that honeybees harbor more viruses than described in the curated HoloBee database [55].

### 3.2. Honey Bee Transcriptome Was Not Heavily Altered by Amygdalin Treatment

Although we found some shifts in microbial and viral levels between treatments and controls, we generally found limited changes in immune gene expression by fold-change. We calculated the fold-change per time point from transformed gene expression count data to discover any large gene expression changes, due to amygdalin consumption, reported as amygdalin treatment relative to the control. We focused on antimicrobial peptides (AMPs), upstream genes in the immunity pathways of Immune deficiency (Imd) and toll, RNA interference (RNAi) genes, and nutrition and behavior genes. None of the assessed immune-system genes, nor the age- and nutrient-sensitive marker vitellogenin or major royal jelly protein 1, showed large fold-change differences between treatment and control samples that were consistent across all time points (Table 2 and Table S4). One gene, annotated as a toll-like receptor Tollo, was found in the top 20 up-regulated genes at the time point ’10 May 2013′ in the amygdalin treatment. The last time point (1 July 2013) seemed to have an overall decrease in gene expression relative to the other three time points in our gene set in the amygdalin treatment (Table 2), except for vitellogenin. Expanding to the top 40 genes with the largest fold-change, we used a Gene Ontology (GO; www.geneontology.org) enrichment analysis, but we found no statistically significant enriched GO terms for the sample points (Table S4). We saw no apparent pattern between gene expression and either bacterial or viral loads.

Lastly, we used the rlog normalized gene expression data to build a principal component analysis (PCA) plot, which showed that most of the variance between samples could be explained by time point rather than by treatment (Figure 3).

## 4. Discussion

We consider our experimental design to be a reasonable simulation of commercial operations. We roughly tested the concentration found in nectar [27]. Presumably, some foragers feeding in almond orchards may be consuming a higher concentration of amygdalin than what we had tested. By the same token, the precise amount of amygdalin that free-flying and nestmate bees consumed remains unknown. However, our results provide insights into how the nectar compound amygdalin may affect the honeybee microbial community and immune gene expression. This includes pathogens that are of critical interest to sustainable agricultural pollination, such as Deformed wing virus and *L. passim,* where we observed lower titers in the amygdalin group compared to control on certain dates. In addition, we did not observe large changes in bacterial titers given our fold-change cutoff.

When we looked for trends of relative loads of bacteria rather than fold-change cutoffs, we consistently observed fewer counts of beneficial *Gilliamella* and *Bifidobacterium* in treated colonies and simultaneously saw increased counts of beneficial *Snodgrassella*. Therefore, our results could suggest that amygdalin may cause slight dysbiosis where *Snodgrassella* replaces *Gilliamella*, the latter possibly being more susceptible to the compound. Consequences of increased *Snodgrassella* could help explain decreased DWV levels, which is something we have observed from our cage feeding studies (Birke, Tauber, and Evans, 2020, in review). However, the loss of an established mutualistic bacterial community could be detrimental to proper immune stimulation, host physiology and pathogen protection [62]. We also saw no apparent pattern relating the relative expression of AMPs with relative bacterial loads, as well as relative virus loads. Therefore, it is plausible that the general change in microbe titers was not related to gene expression of AMPs, for which microbe and virus loads may be dependent on the season and/or plausibly somewhat influenced by the amygdalin treatment.

Indeed, mutualistic bacteria are in the bee’s intestine and may be in direct contact with xenobiotics. However, amygdalin itself does not show antibacterial effects in vitro, and the almond nectar’s bacterial community appears not to be significantly shaped by amygdalin [63]. The effects of amygdalin are plausibly due to its derivative. Beta-glucosidase, a hydrolase enzyme that catalyzes the initial breakdown of amygdalin, is secreted into the mouth from the hypopharyngeal glands of the honeybee, and this enzyme can then be transferred to the midgut [64,65]. The final conversion to toxic cyanide is possible with water. Additionally, the toxicity of amygdalin in bees is supported by the observation that amygdalin ingestion, but not injection, induces abnormal behavior [30]. Therefore, it is probable that the bee is degrading amygdalin into a toxic product when ingested, and there exists a mechanism for controlled chemical absorption in the gut. One hypothesis is that amygdalin’s derivative is toxic to certain gut microbes, although not enough so to decimate the symbionts. How amygdalin is processed in the bee gut or hemolymph and/or by the intestinal microbiota should be a future focus to understand with greater resolution how amygdalin could change the microbial composition.

Viruses are mainly located in the hemocoel, although Sinaivirus may have overall higher titers in gut tissue [66]. But given that amygdalin and plausibly its degraded product can also be found in the hemolymph after oral ingestion [30], albeit at an almost tenfold reduced amount, it is possible that the compound can interact with many viral pools. Another hypothesis is that amygdalin’s derivative is toxic to host cells, thus disrupting viral replication. In Palmer-Young et al. [67], amygdalin-fed honeybees, whether completely reared in bee cages or released back to the colony, had higher levels of hymenoptaecin, lower levels of DWV and no increase in mortality; however, these changes were not statistically significant. In the current study, we used near-natural concentrations of amygdalin, which were five-fold lower than the concentration of amygdalin used in this study by Palmer-Young et al. [67]. That said, the previous experiment complements our current work because, in both instances, DWV titers were lower, albeit not statistically significant, in bees that had consumed amygdalin. Therefore, the decrease in DWV makes our finding even more intriguing. This is because DWV may be considered the most serious of the RNA viral pathogens because this virus, in concert with *Varroa* that transmits the virus, causes deformity, colony loss and reduced individual lifespan [68,69]. Black queen cell virus and Chronic bee paralysis virus were also both consistently lower in the amygdalin group.

We included the Tobacco ringspot virus (TRSV), which is a virus that bees pick up from plants, as a control for the virus group. We used TRSV as a control because there is a lack of sufficient evidence to support that it is a bee pathogen [54]. Interestingly, the titer counts for TRSV between the treatment and control essentially overlapped and followed the same pattern over the two months (relative fold-change was not greater than 2 on any date). This was in contrast to most honeybee-associated viruses. Given the possible lack of evidence that TRSV is adapted to honeybees [54] and replicates in bees [70], despite some debate [71], if we consider that the majority of TRSV present in the bee is not adapted, then our results, which show large viral count differences of honeybee-associated viruses between conditions, suggest that amygdalin was somehow affecting these honeybee viruses that are adapted to bees.

There can be substantial seasonal variation in titers of microbes and viruses. Virus [72], bacteria [73] and *L. passim* [74] presence in bees is typically seasonal. For example, in a year-long survey [72], Lake Sinai Virus was very sparse outside of the spring and summer months of February to July, whereas DWV had the highest titers in the cooler months (September to February). In contrast to these two virus titers, the Black queen cell virus was consistently high over the surveyed months. Pathogens in colonies for commercial almond pollination also have seasonally dependent pathogen prevalence [75]. Pathogen prevalence can also vary by geography and climate [76]. In our current study, total viral loads generally decreased from May to July in all bees, which seemed mostly driven by Sinaivirus, which had the largest abundance. Sinaivirus prevalence decreased from May to July, a similar seasonal swing that was observed in previous work [77], while IAPV and DWV-B were inconsistent across dates. Although Sinaivirus loads in our work did not exactly follow the month-to-month observations in [77], we similarly observed a gradual decrease in Sinaivirus over the summertime. Furthermore, the larger total viral abundance in the amygdalin treatment on June 14 appears driven by both Sinaivirus and Sacbrood virus. Future studies are needed to confirm these survey results as consistent year-to-year observations, as both a survey to understand viral prevalence and to see if amygdalin may affect these viruses under varying temporal circumstances. Although one study found an increase in Sinaivirus in “weak” colonies [66] and despite its large persistence [78], there is a gap in our knowledge on its pathogenicity and disease phenotypes. The uptick of DWV at the last date could be the effect of amygdalin weaning off or from seasonal fluctuations. Altogether, it is difficult to pinpoint a reason. Other studies could analyze freshly dead bees that died due to higher viral loads, which were missed in our work because we only tested living bees, as well as to test colonies from other apiaries that have a different balance of virus richness and evenness. One could also track commercial honeybees as they are moved into and out of almond orchards to observe if symbiont alterations are persistent, and involve both experimental manipulations of amygdalin exposure (as was done here) and testing during a foraging season with plants, such as almonds that contain amygdalin. As noted in [67], repeating compound feeding experiments can yield different results due to such factors as the genetic lineage of bees, season, year, infection level, the experimental setup and molecular techniques [79,80].

Phytochemicals can be harnessed as treatments to improve bee health, and we are currently focusing on natural products as safe and reliable remedies to reduce pathogen loads and/or improve colony health [32]. We are focused on compounds generally recognized as safe (GRAS) to consume, listed by the Federal Food and Drug Administration (FDA) (http://www.accessdata.fda.gov/scripts/fdcc/?set=SCOGS). Amygdalin is not a GRAS compound; however, we felt it was important to test this compound since honeybees have limited foraging options during almond pollination and so are predominately exposed to this compound. In general, both the understanding of forage nectar constituents on bee health and the development of phytochemical applications require an understanding of detrimental and beneficial effects. Many phytochemicals can be deterrents for pollinators or toxic in certain concentrations or contexts, and may have evolved to combat microbes or herbivores [21,81,82,83]. Consumption preferences by concentration appear to be the case for various nectar compounds except for amygdalin [84]. Tiedeken et al. (2014) reported that honeybees do not respond to levels of some compounds, including amygdalin, with a threshold of 10 mmol l^−1^, whereas bumblebees were more sensitive to amygdalin at thresholds of 1 mmol l^−1^ [85]. Generalist bee species may have reduced detection of toxins because they have fewer gustatory receptors. Consequentially, although amygdalin and other nectar phytochemicals may be problematic during individual consumption, their dilution in the colony may make them have negligible effects on the colony, which is important to consider given that the colony is the unit on which eusocial selection acts. In fact, amygdalin consumption in a natural setting may actually be seasonal and due to nectar dearth [27]. Nevertheless, there is a growing body of literature suggesting that phytochemicals in nectars and pollen, even those considered toxic in some cases, can improve pollinator health at certain concentrations by reducing disease. For our work, with no discernable pattern of immune gene expression and microbe and virus titers after ingestion of amygdalin, it is possible that any amygdalin-induced shifts may not overall impact colony health, although future studies are needed to measure whole colony health in this context. We will cautiously approach amygdalin’s effects until we follow colony health metrics over a full commercial operation.

## 5. Conclusions

We fed amygdalin, a secondary compound in the nectar of almond trees, to free-flying honeybees over two months at a natural concentration. Amygdalin treatment appeared to reduce certain microbial and viral titers at specific dates. We did not observe significant changes in honeybee gene expression, due to amygdalin consumption. For future work, one needs to consider the tradeoff of dysbiosis of beneficial symbionts with a reduction in pathogen loads. As is the case with purported mass bee deaths due to linden tree nectar [86], to truly understand the consequences of nectar chemicals on bees, including the holobiont, we need evidence in the context of the floral season, bee biology, reward drivers, total nectar chemistry, as well as cumulative and interactive effects. Natural products remain an intriguing aspect of bee biology, which could influence the livelihood of the backyard and commercial beekeeping.

## Figures and Tables

**Table 1:** Alignment data for microbe and virus counts for each of the four time points and treatments (amygdalin and control). Kraken2 data are a percentage of classified RNA reads (i.e., microbe counts) of all leftover reads after the Hisat2 alignment to the bee genome (“normalization factor” = 1). Hisat2 data are the percentage of RNA reads mapped to the *A. mellifera* genome (further detailed in the Supplementary Information document). When we compare the HoloBee search to the RefSeq search using total viral, total bacterial and total fungal reads, we see comparable values for both virus and bacteria, but a relatively large discrepancy for fungi. We note that the RefSeq database is missing some organisms in the HoloBee database, notably *Nosema*. Overall, we believe that this indicates that our HoloBee-derived search captured most of the expected honeybee-associated microbes.

	10 May 2013		28 May 2013		14 June 2013		1 July 2013	
Alignment/count details	Amygdalin	Control	Amygdalin	Control	Amygdalin	Control	Amygdalin	Control
***A. mellifera* Hisat2 overall alignment rate**	87.08	95.4	89.19	97.06	91.1	97.91	90.99	88.61
**Kraken2-HoloBee total viral reads**	4.30	23.69	7.04	23.65	6.30	10.78	0.64	2.74
**Kraken2-RefSeq total viral reads**	4.66	25.20	7.29	24.36	6.62	11.93	0.68	2.80
**Kraken2-HoloBee total bacterial reads**	0.90	3.14	1.32	6.71	1.23	7.4	1.85	1.40
**Kraken2-RefSeq total bacterial reads**	1.08	3.48	1.49	7.31	1.47	8.14	2.15	1.61
**Kraken2-HoloBee total fungal reads**	0.54	5.20	1.92	10.6	1.21	6.13	0.46	0.54
**Kraken2-RefSeq total fungal reads**	0.07	0.29	0.13	0.55	0.08	0.44	0.07	0.05

**Table 2:** Fold change (FC) for key bee immune-related genes. We chose genes based on immunity [56,57,58,59,60], and also nutrition and behavior [61]. We calculated fold-change by using log_2_FC = log_2_(amygdalin) − log_2_(control), and then FC = 2^log2FC^, where FC > 1 is higher expression and 0-1 is lower expression of amygdalin-treated colonies relative to the control colonies.

		May 10	May 28	June 14	July 1
**Antimicrobial peptides**	Hymenoptaecin	1.02	0.64	2.53	0.53
	Abaecin	1.21	1.17	1.80	0.90
	Apidaecin	1.27	1.25	1.61	0.91
	Defensin-1	0.89	0.82	0.88	0.77
**Upstream toll and Imd/JNK pathways**	Peptidoglycan-recognition protein 1	1.17	0.96	0.99	1.06
	Peptidoglycan recognition protein S2	1.13	1.00	1.07	0.74
	Beta-1,3-glucan-binding protein 1 (gnbp-1)	1.19	1.21	1.22	0.83
	Nuclear factor NF-kappa-B p100 subunit (relish)	1.15	0.94	1.03	0.84
**Hormone (nutrition/behavior)**	Vitellogenin	2.30	0.97	1.23	4.76
	Major royal jelly protein 1	1.057	0.89	0.95	0.83
	Apisimin	1.08	0.94	0.88	0.80
**Other immunity**	Apidermin 3	3.23	3.19	1.73	0.53
	Lysozyme	0.83	0.89	0.70	0.86
**RNAi**	Protein argonaute-2	1.30	1.04	1.15	1.05
	RISC-loading complex subunit TARBP2	1.47	1.03	1.18	0.61

## Supplementary Materials

The following are available online at https://www.mdpi.com/2075-4450/11/11/783/s1, Supplementary Information.
