# Supplementary material for: Colony-Level Effects of Amygdalin on Honeybees and Their Microbes
Source: Insects. 2020 Nov 11;11(11):783. doi: 10.3390/insects11110783 (PMC7698215; doi:10.3390/insects11110783)
Supplement: Supplementary file 1 [file insects-11-00783-s001.zip › Supplement_Revision1/amygdalin-supplement.docx]

Supplement: Colony-level effects of amygdalin on honey bees and their microbes

James P. Tauber^1,#^, Cansu Ö. Tozkar^1,2^, Ryan S. Schwarz^1,3^, Dawn Lopez^1^,  Rebecca E. Irwin^4^, Lynn S. Adler^5^ , Jay D. Evans^1,#^

^1^ Bee Research Laboratory, Beltsville Agricultural Research Center, US Department of

Agriculture, Beltsville, MD 20705, USA

^2^ Yüzüncü Yıl University, Faculty of Agriculture, Department of Agricultural Biotechnology 65000 Van, Turkey

^3^ Department of Biology, Fort Lewis College, 1000 Rim Drive, Durango, CO 81301, USA

^4^ North Carolina State University, Department of Applied Ecology, Raleigh, NC 27695, USA

^5^ University of Massachusetts, Department of Biology, Amherst, MA 01003, USA

#Corresponding Authors: James P. Tauber and Jay D. Evans

Email address: [james.tauber@usda.gov](mailto:james.tauber@usda.gov), jay.evans@usda.gov

**Table of contents**

Nanodrop assessment and pooling of samples for NGS 1

Hisat alignment statistics 5

Size library normalization of count data using log2 9

Before and after rlog transformation of data

(transformed counts used for Figure 1) 10

Information of total RNA samples prior to NGS sequencing.

| **Collection** | **Sample ID** | **Pool** | **(ng /μl)** | **A_260/280_** | **A_260/230_** | **Conversion** | **μl** | **μl** | **Pool** |
| --- | --- | --- | --- | --- | --- | --- | --- | --- | --- |
| 1 | 1 46-A (5\10\13) | A | 1253 | 2.08 | 1.77 | 0.003192338 | 3.192338388 | 6.384676776 | A1 |
| 1 | 1 50-A(5\10\13) | A | 1132.5 | 2.14 | 1.57 | 0.003532009 | 3.53200883 | 7.06401766 | A1 |
| 1 | 1 55-A(5\10\13) | A | 1049.9 | 2.1 | 1.83 | 0.003809887 | 3.809886656 | 7.619773312 | A1 |
| 1 | 1 58-A(5\10\13) | A | 856.9 | 2.12 | 1.52 | 0.004667989 | 4.667989264 | 9.335978527 | A1 |
| 1 | 1 71-A(5\10\13) | A | 846.9 | 2.14 | 1.81 | 0.004723108 | 4.723107805 | 9.44621561 | A1 |
| 1 | 1 73-A(5\10\13) | A | 1087.6 | 2.17 | 1.91 | 0.003677823 | 3.677822729 | 7.355645458 | A1 |
| 1 | 1 47-A(5\10\13) | B | 1260.8 | 2.13 | 1.85 | 0.003172589 | 3.172588832 | 6.345177665 | A2 |
| 1 | 1 54-A(5\10\13) | B | 1037.7 | 2.18 | 1.8 | 0.003854679 | 3.854678616 | 7.709357232 | A2 |
| 1 | 1 56-A(5\10\13) | B | 1049.9 | 2.12 | 1.72 | 0.003809887 | 3.809886656 | 7.619773312 | A2 |
| 1 | 1 64-A(5\10\13) | B | 690.4 | 2.08 | 1.74 | 0.005793743 | 5.793742758 | 11.58748552 | A2 |
| 1 | 1 68-A(5\10\13) | B | 642.2 | 2.13 | 1.71 | 0.006228589 | 6.228589225 | 12.45717845 | A2 |
| 1 | 1 72-A(5\10\13) | B | 1022.1 | 2.09 | 1.69 | 0.003913511 | 3.913511398 | 7.827022796 | A2 |
| 2 | 1 46-C(6\14\13) | A | 792.7 | 2.1 | 1.53 | 0.005046045 | 5.046045162 | 10.09209032 | C1 |
| 2 | 1 50-C(6\14\13) | A | 742.6 | 2.15 | 1.54 | 0.00538648 | 5.386479935 | 10.77295987 | C1 |
| 2 | 1 55-C(6\14\13) | A | 763.1 | 2.06 | 1.64 | 0.005241777 | 5.241776962 | 10.48355392 | C1 |
| 2 | 1 58-C(6\14\13) | A | 813.8 | 2.13 | 1.69 | 0.004915213 | 4.915212583 | 9.830425166 | C1 |
| 2 | 1 71-C(6\14\13) | A | 637.3 | 2.11 | 1.7 | 0.006276479 | 6.276478895 | 12.55295779 | C1 |
| 2 | 1 73-C(6\14\13) | A | 885.6 | 2.14 | 1.84 | 0.004516712 | 4.516711834 | 9.033423668 | C1 |
| 2 | 1 47-C(6\14\13) | B | 470.8 | 2.07 | 1.82 | 0.008496177 | 8.49617672 | 16.99235344 | C2 |
| 2 | 1 54-C(6\14\13) | B | 860.2 | 2.13 | 1.57 | 0.004650081 | 4.650081376 | 9.300162753 | C2 |
| 2 | 1 56-C(6\14\13) | B | 1052.6 | 2.09 | 1.81 | 0.003800114 | 3.800114003 | 7.600228007 | C2 |
| 2 | 1 64-C(6\14\13) | B | 815.5 | 2.16 | 1.65 | 0.004904966 | 4.904966278 | 9.809932557 | C2 |
| 2 | 1 68-C(6\14\13) | B | 787.9 | 2.11 | 1.66 | 0.005076786 | 5.076786394 | 10.15357279 | C2 |
| 2 | 1 72-C(6\14\13) | B | 858.4 | 2.11 | 1.9 | 0.004659832 | 4.659832246 | 9.319664492 | C2 |
| 3 | 1 46-B(5\28\13) | A | 677.3 | 2.02 | 1.4 | 0.005905802 | 5.905802451 | 11.8116049 | B1 |
| 3 | 1 50-B(5\28\13) | A | 864.4 | 2.15 | 1.48 | 0.004627487 | 4.627487274 | 9.254974549 | B1 |
| 3 | 1 55- B(5\28\13) | A | 910.7 | 2.11 | 1.47 | 0.004392226 | 4.39222576 | 8.784451521 | B1 |
| 3 | 1 58- B(5\28\13) | A | 815.5 | 2.18 | 1.57 | 0.004904966 | 4.904966278 | 9.809932557 | B1 |
| 3 | 1 71- B(5\28\13) | A | 673.1 | 2.1 | 1.55 | 0.005942653 | 5.942653395 | 11.88530679 | B1 |
| 3 | 1 73- B(5\28\13) | A | 765.4 | 2.18 | 1.68 | 0.005226026 | 5.226025608 | 10.45205122 | B1 |
| 3 | 1 47- B(5\28\13) | B | 782.3 | 2.08 | 1.56 | 0.005113128 | 5.113127956 | 10.22625591 | B2 |
| 3 | 1 54- B(5\28\13) | B | 922.8 | 2.07 | 1.67 | 0.004334634 | 4.334633723 | 8.669267447 | B2 |
| 3 | 1 56- B(5\28\13) | B | 1365.9 | 2.12 | 1.52 | 0.002928472 | 2.92847207 | 5.856944139 | B2 |
| 3 | 1 64- B(5\28\13) | B | 894 | 2.08 | 1.48 | 0.004474273 | 4.474272931 | 8.948545861 | B2 |
| 3 | 1 68- B(5\28\13) | B | 861.1 | 2.06 | 1.45 | 0.004645221 | 4.645221229 | 9.290442457 | B2 |
| 3 | 1 72- B(5\28\13) | B | 835.4 | 2.17 | 1.56 | 0.004788125 | 4.788125449 | 9.576250898 | B2 |
| 4 | 1 46- 1 B(7/1/13) | A | 1145 | 2.14 | 1.46 | 0.00349345 | 3.493449782 | 6.986899563 | D1 |
| 4 | 1 50- 1 B(7/1/13) | A | 870.7 | 2.2 | 1.66 | 0.004594005 | 4.594004824 | 9.188009647 | D1 |
| 4 | 1 58- 1 B(7/1/13) | A | 778.8 | 2.16 | 1.6 | 0.005136107 | 5.136106831 | 10.27221366 | D1 |
| 4 | 1 71- 1 B(7/1/13) | A | 719.8 | 2.08 | 1.57 | 0.005557099 | 5.557099194 | 11.11419839 | D1 |
| 4 | 1 73- 1 B(7/1/13) | A | 785.5 | 2.17 | 1.77 | 0.005092298 | 5.092297899 | 10.1845958 | D1 |
| 4 | 2 55- BB(7/1/13) | A | 850.3 | 2.15 | 1.69 | 0.004704222 | 4.704222039 | 9.408444079 | D1 |
| 4 | 1 47- 1 B(7/1/13) | B | 636.8 | 2.01 | 1.51 | 0.006281407 | 6.281407035 | 12.56281407 | D2 |
| 4 | 1 54- 1 B(7/1/13) | B | 757.5 | 2.15 | 1.74 | 0.005280528 | 5.280528053 | 10.56105611 | D2 |
| 4 | 1 56- 1 B(7/1/13) | B | 822.3 | 2.13 | 1.7 | 0.004864405 | 4.864404718 | 9.728809437 | D2 |
| 4 | 1 64- 1 B(7/1/13) | B | 795.4 | 2.16 | 1.79 | 0.005028916 | 5.028916269 | 10.05783254 | D2 |
| 4 | 1 68- 1 B(7/1/13) | B | 784.8 | 2.1 | 1.82 | 0.00509684 | 5.096839959 | 10.19367992 | D2 |
| 4 | 1 72- 1 B(7/1/13) | B | 911.1 | 2.16 | 1.63 | 0.004390297 | 4.390297443 | 8.780594885 | D2 |

**Hisat2 alignment**

A1

47215090 reads; of these:

47215090 (100.00%) were paired; of these:

6548275 (13.87%) aligned concordantly 0 times

13311451 (28.19%) aligned concordantly exactly 1 time

27355364 (57.94%) aligned concordantly >1 times

----

6548275 pairs aligned concordantly 0 times; of these:

21149 (0.32%) aligned discordantly 1 time

----

6527126 pairs aligned 0 times concordantly or discordantly; of these:

13054252 mates make up the pairs; of these:

12197924 (93.44%) aligned 0 times

467929 (3.58%) aligned exactly 1 time

388399 (2.98%) aligned >1 times

87.08% overall alignment rate

A2

35966651 reads; of these:

35966651 (100.00%) were paired; of these:

2022894 (5.62%) aligned concordantly 0 times

11338073 (31.52%) aligned concordantly exactly 1 time

22605684 (62.85%) aligned concordantly >1 times

----

2022894 pairs aligned concordantly 0 times; of these:

18860 (0.93%) aligned discordantly 1 time

----

2004034 pairs aligned 0 times concordantly or discordantly; of these:

4008068 mates make up the pairs; of these:

3305556 (82.47%) aligned 0 times

381700 (9.52%) aligned exactly 1 time

320812 (8.00%) aligned >1 times

95.40% overall alignment rate

B1

42736336 reads; of these:

42736336 (100.00%) were paired; of these:

5064575 (11.85%) aligned concordantly 0 times

10930622 (25.58%) aligned concordantly exactly 1 time

26741139 (62.57%) aligned concordantly >1 times

----

5064575 pairs aligned concordantly 0 times; of these:

15042 (0.30%) aligned discordantly 1 time

----

5049533 pairs aligned 0 times concordantly or discordantly; of these:

10099066 mates make up the pairs; of these:

9238924 (91.48%) aligned 0 times

411914 (4.08%) aligned exactly 1 time

448228 (4.44%) aligned >1 times

89.19% overall alignment rate

B2

37277868 reads; of these:

37277868 (100.00%) were paired; of these:

1442125 (3.87%) aligned concordantly 0 times

10617184 (28.48%) aligned concordantly exactly 1 time

25218559 (67.65%) aligned concordantly >1 times

----

1442125 pairs aligned concordantly 0 times; of these:

14442 (1.00%) aligned discordantly 1 time

----

1427683 pairs aligned 0 times concordantly or discordantly; of these:

2855366 mates make up the pairs; of these:

2192615 (76.79%) aligned 0 times

333632 (11.68%) aligned exactly 1 time

329119 (11.53%) aligned >1 times

97.06% overall alignment rate

C1

44315354 reads; of these:

44315354 (100.00%) were paired; of these:

4454933 (10.05%) aligned concordantly 0 times

12436668 (28.06%) aligned concordantly exactly 1 time

27423753 (61.88%) aligned concordantly >1 times

----

4454933 pairs aligned concordantly 0 times; of these:

20349 (0.46%) aligned discordantly 1 time

----

4434584 pairs aligned 0 times concordantly or discordantly; of these:

8869168 mates make up the pairs; of these:

7890884 (88.97%) aligned 0 times

513359 (5.79%) aligned exactly 1 time

464925 (5.24%) aligned >1 times

91.10% overall alignment rate

C2

49464063 reads; of these:

49464063 (100.00%) were paired; of these:

1521004 (3.07%) aligned concordantly 0 times

14576290 (29.47%) aligned concordantly exactly 1 time

33366769 (67.46%) aligned concordantly >1 times

----

1521004 pairs aligned concordantly 0 times; of these:

21526 (1.42%) aligned discordantly 1 time

----

1499478 pairs aligned 0 times concordantly or discordantly; of these:

2998956 mates make up the pairs; of these:

2070242 (69.03%) aligned 0 times

503518 (16.79%) aligned exactly 1 time

425196 (14.18%) aligned >1 times

97.91% overall alignment rate

D1

39864039 reads; of these:

39864039 (100.00%) were paired; of these:

4006226 (10.05%) aligned concordantly 0 times

10918354 (27.39%) aligned concordantly exactly 1 time

24939459 (62.56%) aligned concordantly >1 times

----

4006226 pairs aligned concordantly 0 times; of these:

15639 (0.39%) aligned discordantly 1 time

----

3990587 pairs aligned 0 times concordantly or discordantly; of these:

7981174 mates make up the pairs; of these:

7185073 (90.03%) aligned 0 times

391260 (4.90%) aligned exactly 1 time

404841 (5.07%) aligned >1 times

90.99% overall alignment rate

D2

41383816 reads; of these:

41383816 (100.00%) were paired; of these:

5069227 (12.25%) aligned concordantly 0 times

11214451 (27.10%) aligned concordantly exactly 1 time

25100138 (60.65%) aligned concordantly >1 times

----

5069227 pairs aligned concordantly 0 times; of these:

17112 (0.34%) aligned discordantly 1 time

----

5052115 pairs aligned 0 times concordantly or discordantly; of these:

10104230 mates make up the pairs; of these:

9425925 (93.29%) aligned 0 times

380191 (3.76%) aligned exactly 1 time

298114 (2.95%) aligned >1 times

88.61% overall alignment rate


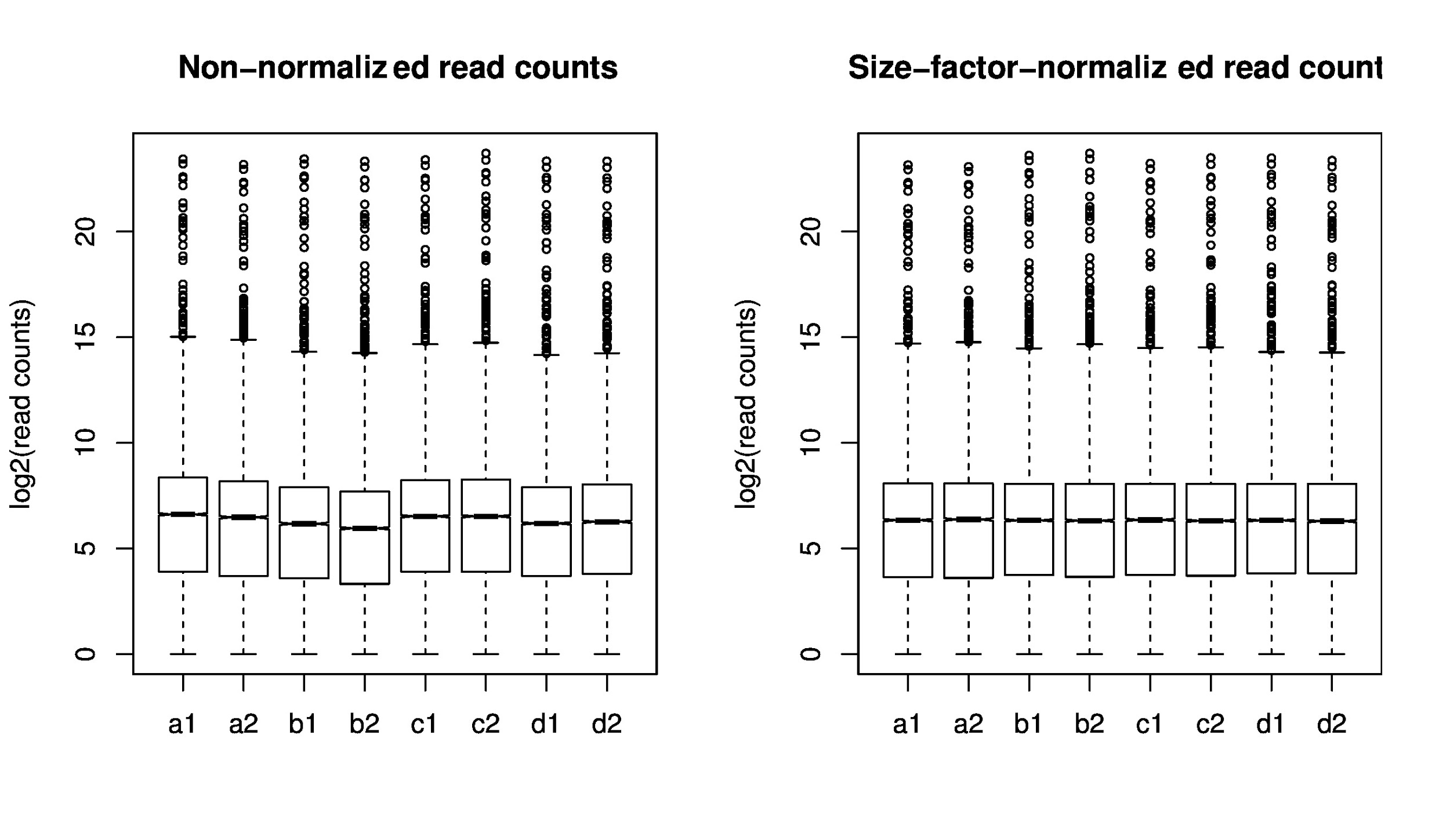


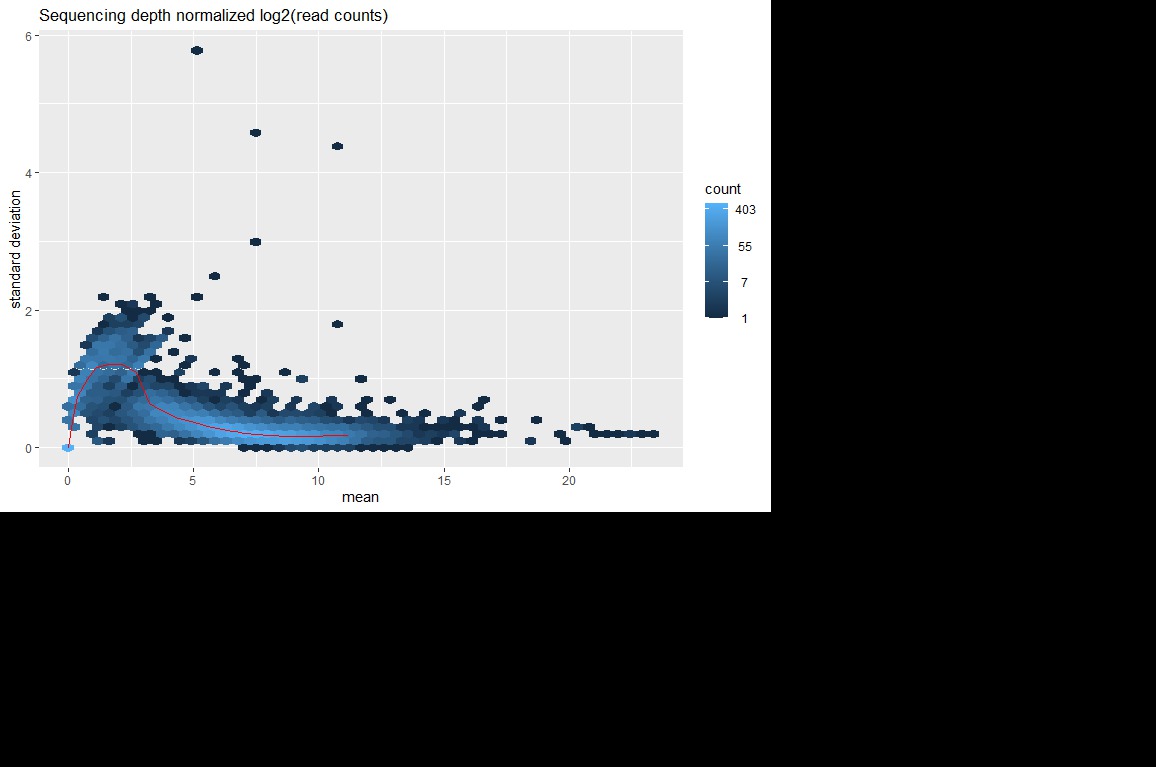


Before rlog transformation of all the count data in DESeq2.


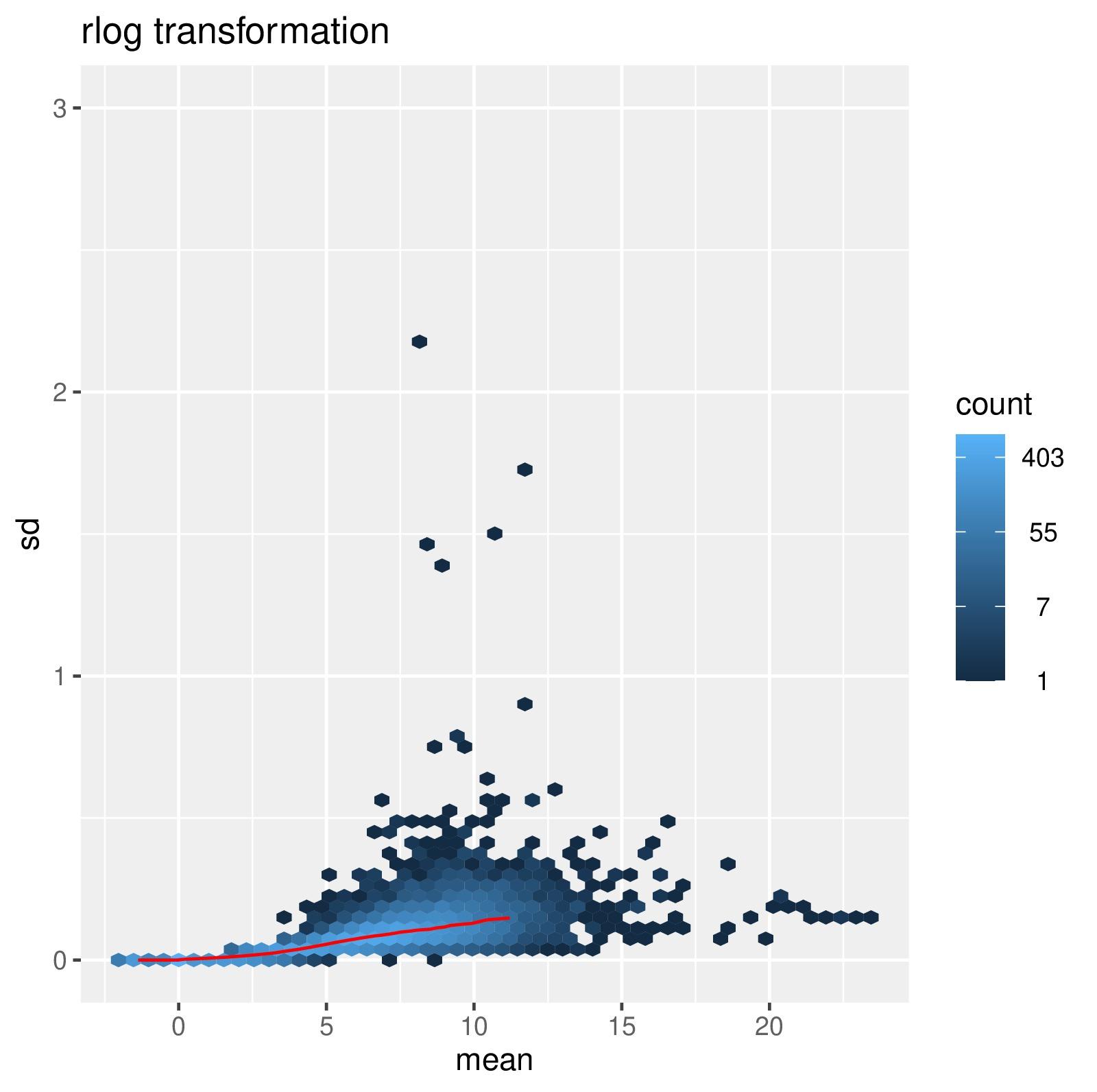


After rlog transformation of all count data in DESeq2.
